# 50 Hz Temporal Magnetic Field Monitoring from High-Voltage Power Lines: Sensor Design and Experimental Validation

**DOI:** 10.3390/s24165325

**Published:** 2024-08-17

**Authors:** Kenneth Deprez, Tom Van de Steene, Leen Verloock, Emmeric Tanghe, Liesbeth Gommé, Mart Verlaek, Michel Goethals, Karen van Campenhout, David Plets, Wout Joseph

**Affiliations:** 1 Department of Information Technology, Ghent University/imec WAVES, 9052 Ghent, Belgium; 2 Department of Environment & Spatial Development, Flemish Planning Bureau for the Environment and Spatial Development, 1000 Brussels, Belgium

**Keywords:** extremely low frequency (ELF), electromagnetic field (EMF), magnetic field exposure sensor, monitoring sensor

## Abstract

A low-cost, tri-axial 50 Hz magnetic field monitoring sensor was designed, calibrated and verified. The sensor was designed using off-the-shelf components and commercially available coils. It can measure 50 Hz magnetic fields originating from high-voltage power lines from 0.08 µT to 364 µT, divided into two measurement ranges. The sensor was calibrated both on-board and in-lab. The on-board calibration takes the circuit attenuation, noise and parasitic components into account. In the in-lab calibration, the output of the developed sensor is compared to the benchmark, a narrowband EHP-50. The sensor was then verified in situ under high-voltage power lines at two independent measurement locations. The measured field values during this validation were between 0.10 µT and 13.43 µT, which is in agreement with other reported measurement values under high-voltage power lines in literature. The results were compared to the benchmark, for which average deviations of 6.2% and 1.4% were found, at the two independent measurement locations. Furthermore, fields up to 113.3 µT were measured in a power distribution sub-station to ensure that both measurement ranges were verified. Our network, four active sensors in the field, had high uptimes of 96%, 82%, 81% and, 95% during a minimum 3-month interval. In total, over 6 million samples were gathered with field values that ranged from 0.08 µT to 45.48 µT. This suggests that the proposed solution can be used for this monitoring, although more extensive long-term testing with more sensors is required to confirm the uptime under multiple circumstances.

## 1. Introduction

Increasing electrification and decentralized power production require reinforcement of the high-voltage grid. Currently, most electrical energy is distributed by means of power lines. Whilst essential for delivering electricity, power lines generate electromagnetic fields at extremely low frequencies (ELF). This exposure to ELF has raised concerns in society about the possible health impact of ELF radiation for many decades and still persists.

A statistical relationship was found between chronic magnetic field exposure beyond 0.4 µT and childhood leukemia [1]. However, a causal relationship is lacking. A multitude of studies have explored the correlation between potential health effects and long-term exposure to magnetic fields originating from power lines [2,3,4,5,6,7,8,9,10]. To determine long-term exposure levels and ensure compliance with established safety standards, continuous monitoring of these magnetic field levels is essential. This is furthermore a crucial part of Flemish policy, the region in which the tests in this work will be performed.

The International Commission on Non-Ionizing Radiation Protection (ICNIRP) has published two guidelines regarding ELF magnetic fields [1,11]. In 1998, they set a general public reference level of 100 µT for magnetic fields at 50 Hz, also adopted by the European Union (EU) and the Flemish government. In 2010, this was revised by ICNIRP to 200 µT, with the occupational reference level being five times higher. In Flanders and the EU, the reference level is still 100 µT [12].

Magnetic field exposure is usually quantified either via spot measurements or via personal exposure measurements [13,14,15,16,17,18,19,20,21,22,23,24,25,26,27,28,29,30,31,32,33]. Spot measurements entail that magnetic fields are measured at a specific place during a short time interval. These measurements might be conducted periodically, but cannot be regarded as continuous. Personal exposure measurements on the other hand use exposimeters attached to the body to measure the magnetic field. These are often used for a short period of time and are mostly related to the subjects specific work environments. It has been shown that the results are influenced by the test subject and the position of the personal exposimeter with regard to the source. A recent survey [14] summarizes all research efforts from 2015 to 2023 about ELF electric and magnetic field exposure assessment. However, no real long-term measurements are presented in this work, further underwriting the need for an ELF monitoring network.

Hence, new measurement methods must be incorporated to enable continuous monitoring at fixed locations, driven by both the need for public information and the lack of such knowledge in the scientific community. The goal of this research is to design and validate a low-cost ELF sensor for long-term monitoring of 50 Hz magnetic fields originating from high-voltage power lines and analyze its measured fields over time. By analyzing the data obtained from monitoring sensors at different locations, spatial and temporal variations of magnetic fields can be determined, contributing to a better understanding of their potential impact on human health and the environment. Furthermore, this would complement existing telecommunication monitoring networks [34,35,36,37,38,39,40], yielding a complete characterization of the electromagnetic field exposure from 50 Hz to 6 GHz.

The novelty of this paper is as follows: (1) design of an ELF sensor with a broad measurement range, allowing measuring both the EU limit of 100 µT and the chronic low-intensity level of 0.4 µT accurately. (2) 24-h validation of the sensor, (3) deployment of an experimental online monitoring network over several months, gathering a very large dataset of over 6 million samples with a high resolution, enabling long-term ELF exposure assessment. To the authors’ knowledge, this is the first time a measurement network is designed for magnetic ELF in this proportion.

## 2. Materials and Methods

### 2.1. Current Existing Sensors

Current commercially available sensors and measurement probes for ELF measurements are summarized in Table 1.

The EHP-50 from Narda Safety Test Solutions GmbH, Pfullingen, Germany uses Fast Fourier Transform (FFT) for a broad frequency range. The sensor can either be placed in stand-alone mode, in which it can measure up to 24 h, or the magnetic field strength can be read immediately from an attached computer through the specific designed software. However, this sensor has no ability to communicate the measured field strengths, nor to lengthen the data logging. Additionally, the price is high. Due to the high sensitivity and range, this sensor will be used as the “golden standard” in testing and calibration.

The ELT-400 from Narda Safety Test Solutions GmbH, Pfullingen, Germany is a hand-held device in which a display is foreseen to instantly read the magnetic field strength. There is a high acquisition cost and it is not weatherproof. It would not be feasible to build a network with these sensors, but it can be used when placing the sensors for the monitoring network as it can locate a local maximum.

The other sensors listed in the table are hand-held devices. However, none of these are readily usable for long-term, online monitoring of magnetic field values. None of the above sensors have an IoT communication protocol built in. Furthermore, these were mostly designed to measure the magnetic field exposure over a short period of time instead of continuously. The final row of the table outlines the minimum specifications for a long-term magnetic field exposure sensor, as will be discussed in depth in Section 2.2.1. The sensor should operate within a frequency range of 10 Hz to 300 Hz and measure across three axes. It must be capable of detecting magnetic fields from 0.1 µT to 200 µT. Additionally, the cost should be a fraction of the EHP-50, to allow for an extensive deployment within a limited budget.

### 2.2. Circuit Design

#### 2.2.1. Requirements

The objective of this study is to design a sensor capable of measuring magnetic fields originating from power lines. The first requirement specifies that the frequency range in which the sensor should operate is 10–300 Hz, with a focus on 50 Hz as these sensors will be placed in Flanders, Belgium. The second requirement is that the sensor must be able to transmit the measured field values through a data platform to enable real-time monitoring. Third, the sensor should be capable of long-term operation (i.e., >1 year) and have a high uptime during this period. Additionally, the cost should be kept low to enable widespread deployment within a constrained budget.

Fourth, the measurement uncertainty and accuracy must be adequate to measure field strengths of 0.4 µT and 100 µT, corresponding to long-term chronic low-intensity fields reported in [1] and the European recommendation that follows [11] and is focused upon in Flemish policy. The 100 µT value represents an acute exposure limit, while Flanders aims for an annual average exposure below 0.4 µT. A minimal accuracy of 4 nT must be achieved for fields around 0.4 µT. For higher fields (>30 µT), an accuracy of 0.1 µT is sufficient. Fifth, the sensor must transmit the measured magnetic field levels at least every 5 s, to provide high-resolution temporal exposure data. The sensor must measure the three orthogonal components to obtain the total magnetic field. Finally, the sensors must be weatherproof, as they will be installed predominantly outdoors, in the vicinity of power lines.

#### 2.2.2. Circuit Exploration

In this research, a Printed Circuit Board (PCB) is designed, which consists of an active bandpass filter, analog to digital converter (ADC), and a microcontroller to read, analyze and send the data. The signal that must be processed is captured by three magnetic coils, connected through connectors with the PCB.

As mentioned, the considered source is high-voltage power lines. The current through these power lines generates a time-varying magnetic field *B*. By Faraday’s law of induction, the magnetic field *B* induces an electric field *E*, as expressed by

(1) $$\nabla \times E=-\frac{\partial B}{\partial t}.$$

Ampère’s Law (with Maxwell’s correction) relates the magnetic field H to the current density J and the time-varying electric displacement field D, given by

(2) $$\nabla \times H=J+\frac{\partial D}{\partial t}.$$

The coils detect the magnetic field by measuring the voltage induced in the coil. For a coil with N turns and surface area A, the induced electromotive force E is given by

(3) $$E=-N\frac{d\Phi_{B}}{dt}$$

where $\Phi_{B}=\int_{S}B\cdot dA$ is the magnetic flux through the coil. This induced voltage is then processed by the proposed PCB. Ref. [41] provides additional in-depth theoretical analysis of coil sensors and discusses the design of such a coil.

The designed circuit must be low-cost and use standard components to ensure continuity. The captured signal is processed on the PCB. First, a first order high pass filter was designed using a RC circuit connected to an operational amplifier (opamp). Then, a second order active low pass filter was designed, again using a RC circuit connected to an opamp. An amplifying stage was added between the filtering stages, increasing the Signal-to-Noise Ratio (SNR) as possible low-frequency noise has already been filtered by the high pass filter.

**Figure 1:**
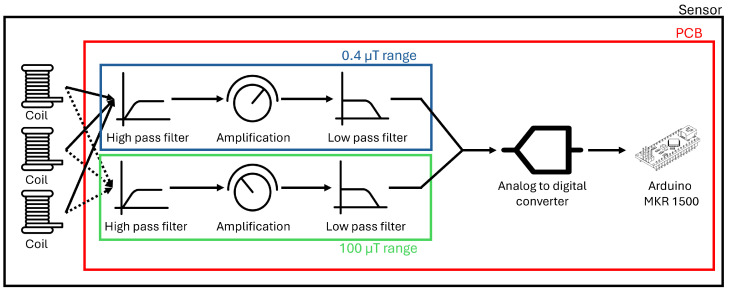
Schematic overview of the full sensor. Three coils are connected to two parallel filtering stages. The signal is then passed through the ADC, before being sent to a database by the Arduino.

Figure 1 summarizes the PCB and sensor design. It indicates the stages of the PCB (red rectangle) and the total sensor (black rectangle). Each coil is connected to the filtering stage of the PCB. Due to the high dynamic range, two measurement ranges are defined. This entails that the PCB has two parallel filtering stages, but with a different amplification. The captured signal by the coils is thus passed through two parallel circuits, in which only the amplification stage is different. The first of the parallel circuits focuses on the 0.4 µT range, whilst the second focuses on the higher field values (100 µT range).

To protect the components from a voltage peak, zener diodes were added into the feedback loop of the high-pass filter. The zener diode limits the peak voltage to the input voltage, protecting both parallel circuits.

For this research, a 16-bit delta-sigma ADC with a low noise level and a theoretical sample rate of 153.6 kilo Samples Per Second (kSPS) is used. Additionally, the ADC has a programmable amplification of 0.33× to 64×, so the range of the ADC can be tailored specifically to the circuit. Up to 8 channels can be connected, 6 channels are used by the current design.

We opted for a microcontroller with mobile internet properties, to ensure a more flexible rollout of the ELF sensors. The Arduino MKR NB 1500 was chosen for this circuit. This microcontroller has an uBlox SARA-R410M-02B chip, which provides mobile telecommunication possibilities. The microcontroller can use GPRS, LTE-M and NB-IoT networks, which can be found in most of Asia, Europe, and North and South America. The Arduino is equipped with a connector to attach an RF antenna to communicate. For this research, a broadband antenna is used.

Lastly, the coil is discussed. The measurement range of the used coil should have a similar range as the sensor, with the peak resonance around 50 Hz. Later, the sensor could be adapted to measure 60 Hz if needed. The used coil is a cylindric, ferromagnetic-core magnetic coil bought from Magnetic Sciences (MC858). This coil (70 mm × 22 mm) has a frequency range of 10–400 Hz, with a resonance peak between 30 Hz and 100 Hz. The number of turns and area is not specified by the manufacturer. The coil produces an output voltage calibrated to a continuous sine wave magnetic field strength. Each sensor has a NIST traceable calibration certificate. Furthermore, the coil can measure up to 5 mT. Other alternatives were considered, but this was the best choice considering the cost, measurement range and reactivity.

**Figure 2:**
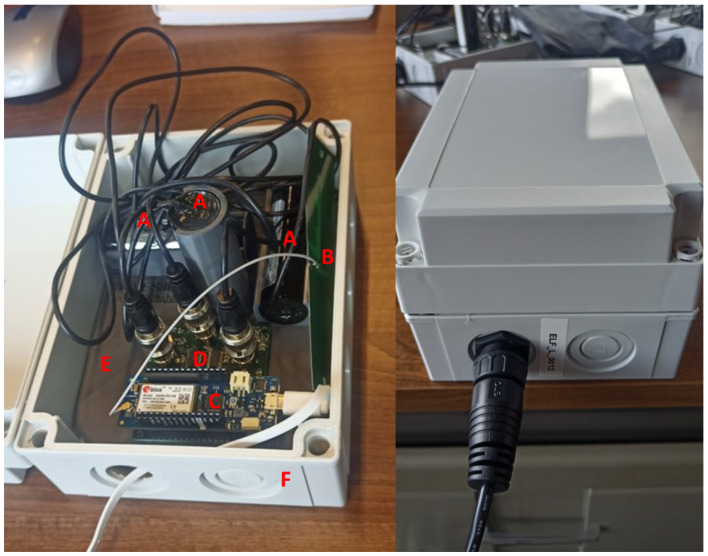
Overview of full sensor. (A) three orthogonally placed magnetic coils; (B) RF antenna for communication; (C) Arduino MKR NB 1500; (D) ELF sensor PCB; (E) 3D print; (F) weatherproof box.

The circuit measures all three orthogonal components simultaneously to capture the entire magnetic field. Hence, each coil is connected to two parallel filtering circuits, indicated by Figure 1. This has led to a final design after performing the verification, shown in Figure 2. To verify and calibrate the full sensor, the three coils must be orthogonally placed with regard to each other. Furthermore, the sensor must be weatherproof. Hence, a 3D printed holder is inserted into an International Protection (IP) rated box, which can be seen in Figure 2.

The sensor is connected to the mains power by a 5 V DC adapter. Hence, the power cable does not influence the sensor. The sensor has an internal sample rate of 2000 Hz. Data is averaged over 5 s (i.e., 10,000 samples) and corresponds to the output voltage of the ADC. The sensor sends these averaged values to a backend each 5 s by means of an MQTT protocol. In total, six values are sent (i.e., two values per orthogonal component in correspondence with the two measurement ranges).

### 2.3. Verification and Calibration

Verification and calibration are performed on the designed PCB. A signal generator (PSG9080) and digital oscilloscope (Picoscope 3406) are used. As verification, a known signal is inserted into the PCB and measured with the oscilloscope after each filtering stage to ensure a correct propagation of the signal throughout the circuit. The filters and amplification are verified with two tests. The first test consisted of a frequency sweep of 1 Hz to 10 kHz. The second test consisted of an amplitude sweep to verify the response of the circuit. The goal of the calibration is to link the output of the PCB (voltage) to a magnetic field exposure level.

The calibration is split into two tests.

**Figure 3:**
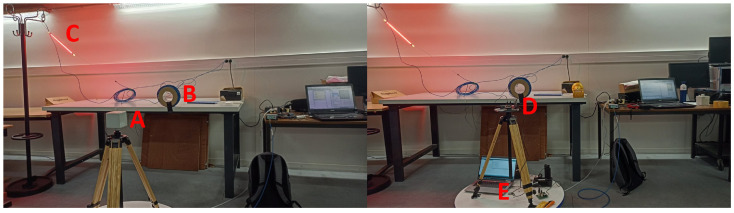
In-lab calibration. (A) EHP-50; (B) 50 Hz source; (C) heat lamp; (D) developed sensor; (E) turntable.

(1) An on-board calibration is performed. An amplitude sweep on a fixed frequency (50 Hz) is sent into the board, the output is captured by the microcontroller. Then, the output is compared to the input and is stored in a look-up-table (LUT). This calibration takes the circuit attenuation, noise and parasitic components into account.
(2) An in-lab calibration is performed. The full sensor (coils + PCB) is calibrated by rotating the sensor twice while a dominant, 50 Hz source is nearby. The source in this case is a heat lamp of 1.8 kW with a twisted 20 m coil (circumference: 20 cm) to strengthen the field (Figure 3). The sensor was fixed at the same height as the coil on a wooden three-piece, which was standing on a turntable. The turntable rotated at 2 degrees per second, first clockwise, then counterclockwise. The entire calibration lasts 6 min. The rotation was performed to take the location dependence of the coils into account, as these could not all be centered in the sensor. A maximal linear displacement of 6 cm of the coils is obtained due to the rotation.

The result is compared to the EHP-50, which is regarded as the benchmark. This is repeated for at least two distances from the source. This calibration factor (deviation with regard to EHP-50) is added to the LUT to obtain a fully calibrated system that can be placed in the field. An additional calibration can be performed at the location of installation to compensate for environmental factors (e.g., installed next to a metal surface, or next to a wall).

Typically, the sensor would be installed in the vicinity of a high-voltage power line. The sensor is weatherproof, so it can be installed both indoors and outdoors. The only requirement is that an outlet must be available. Whenever possible, the sensor should be installed on the top floor, where the distance to the source is as low as possible, so that the realistic maximum values can be measured. They can also be installed where people spend most of their time so that the measured field values give a realistic indication of the total human exposure.

## 3. Results

### 3.1. Circuit Design and Sensor Design

The designed high pass filter has a cutoff frequency of 9.65 Hz, while the second order low pass filter has a cutoff frequency of 318.31 Hz, for both measurement ranges. Furthermore, a software filter is implemented. The microcontroller performs a Fourier analysis, focusing solely on the 50 Hz component to minimize computational costs with a sample frequency of 1000 Hz and a sample period of 5 s.

Assuming an input magnetic field $B\left(t\right)=B_{0}*cos\left(wt\right)$, with w the target angular frequency of 2∗π∗50 Hz, a purely sinusoidal signal $B_{o}*cos\left(wt\right)$ will yield a measurement result of $B_{o}$. A superimposed signal of 150 Hz, the first harmonic on this original signal, will not change the amplitude of the 50 Hz Fourier coefficient since an integer number of 150 Hz periods fit in the measurement window of 5 s. Only frequencies very close to 50 Hz and aliasing frequencies of 50 Hz +k∗1000 Hz will have a small influence on the measurement, due to the finite measurement window of 5 s and finite sampling frequency, respectively.

This approach is suitable for monitoring high-voltage power lines, where a dominant 50 Hz signal (in Europe) is expected. For other use cases requiring a broader frequency range, the Fourier analysis can be omitted to match the hardware capabilities.

A frequency sweep and amplitude sweep were performed on the design as described in Section 2.3. These indicated that the sensor could detect 50 Hz signals. Hence, the coils were connected to the PCB to verify this with real 50 Hz sources (e.g., laptop charger, solar panel inverter). This verification served as a simple test to ensure that the system could detect changes in 50 Hz signals, no quantifying of the measured field was performed.

After the positive verification, a calibration was performed. First, both measurement ranges are defined. The measurement ranges rely on the amplification in the feedback loop of the high-pass filter. For the 0.4 µT measurement range, an amplification of factor 2 was added to the circuit. The manufacturer calibrated the magnetic coils by placing them in a known magnetic field at specific frequencies and measuring the sensor output voltage in Volts per Gauss (V/G). For the coil model MC858, as used in this work, the output voltage at 50 Hz (calibrated sensor response) is 2.352 V/G or 23.52 mV/µT. The bit response for a single axis is calculated as follows:

(4) $$\frac{Maximum\;voltage}{available\;bit\;range}=\frac{3300\mathrm{mV}}{2^{16}-1}=0.101\mathrm{mV}$$

(5) $$F\left(T\right)=\frac{\mathrm{mV}}{Calibrated\;sensor\;response*PCB\;amplification}=\frac{0.101\mathrm{mV}}{47.04\mathrm{mV}/\mu T}=0.002\;\mu T$$

Although a 16-bit ADC was used, only a 15-bit range is used due to reference point, which is half the maximum voltage. Hence, only the positive range of the ADC is used. For the current parameters in the 0.4 µT, a single axis has a minimal step response of 2 nT and a realistic maximum of 34 µT. However, the sensor uses three orthogonal axis, the maximum field value that can be measured is then 58 µT. Higher values will lead to saturation of the sensor. The minimal step does not give an indication of the lowest measurable field strength, as this is dependent on other factors (e.g., noise) and was determined to be 0.08 µT in testing.

Similar calculations are performed for the 100 µT measurement range for the three axes. For this range, an attenuation of factor 3 was used in the feedback loop. Hence, the minimal step response of a singular axis is 13 nT and the realistic maximum field level that can be measured with the three coils and without saturation is 364 µT. The total measurable magnetic field is thus 0.08 µT to 364 µT, divided into two measurement ranges. This measurement range is extensive and comparable with the other low-cost sensors listed in Table 1.

A sensor is designed according to the minimal requirements listed in Table 1. The sensor operates within a frequency range of 9 Hz to 318 Hz, with Fourier analysis applied to focus on 50 Hz. It features three perpendicular axes for magnetic field measurement. The magnetic range exceeds the minimal requirements, capable of detecting magnetic fields from 0.08 µT to 364 µT. The cost of the sensor is minimized to fit within the price range defined in Table 1, making it significantly cheaper than the baseline (EHP-50) and less expensive than the EMDEX II, though more costly than the handheld sensors listed in rows 5 to 8. Additionally, the developed sensor includes IoT connectivity via wireless telecommunication technology, is weatherproof and supports long-term data logging.

### 3.2. Calibration

In total, 12 sensors were calibrated.

**Figure 4:**
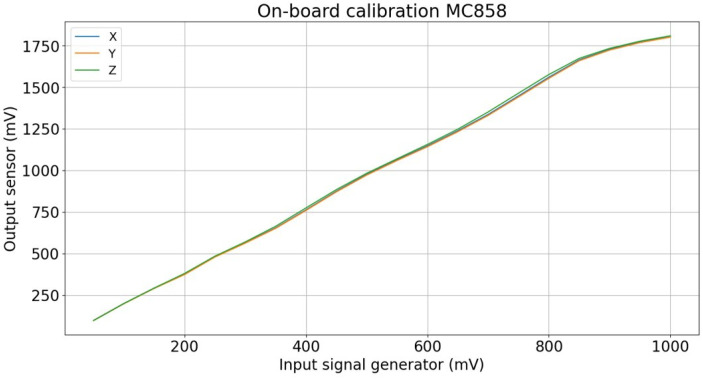
On -board calibration. Three orthogonal axes were sequentially calibrated.

For the on-board calibration, the response of the sensor to a known input voltage was measured. Figure 4 shows the input voltage versus the output voltage for the low-range part of the circuit. The input voltage was incremented by 50 mV for each measurement point. The three axes (i.e., orthogonal components x, y and z) were measured sequentially. However, due to the identical components used, an equal response is expected and obtained (i.e., the correlation between the output signals is 1). A linear response is obtained for an input voltage of 50 mV to 825 mV (x-axis), which has doubled at the output (y-axis). From 1650 mV (y-axis) on, the sensor saturates. Saturation occurs when the input voltage exceeds the maximum voltage of the zener diodes (3.3 V).

A similar response is obtained for the high-range section of the circuit. However, the output voltage is now three times lower than the input voltage. Thus, when an input voltage of 600 mV is inserted, an output voltage of 200 mV is obtained.

An LUT was constructed for the sensor by means of interpolation between the 50 mV measurement points. This interpolation is performed for all calibrated sensors. The twelve sensors were compared and the average standard deviation over the three channels and the full measurement range was only 0.3%. The maximum standard deviation was 0.5%. It can be concluded that the 12 calibrated sensors all had an identical response to the input voltage and the global calibration table, based on the average over the twelve sensors, can be constructed and used. This could lead to a decrease in the total cost of the sensor in the future, as not all sensors would need to be individually calibrated. Only a few sensors should be randomly tested.

The in-lab calibration was performed as discussed in Section 2.3. Table 2 summarizes the obtained results. The sensors were placed at two distances from the source. Here, only the results for the 0.4 µT range are shown, as the magnetic field values measured fit perfectly within this range (0.08 µT to 58 µT).

For the in-lab calibration, the sensors obtained an average underestimation of 36.7% for both measurement ranges with regard to the EHP-50, which is considered as the baseline. There is an average variation of 0.7% between the twelve sensors, with a maximum variation of 1.5% for both tests. This variation can be induced due to the global calibration table, the slight variability in the components, and the placement of these components and the manual placing of the sensors in front of the dominant source and the slight variability in the current through the source. Furthermore, an average calibrated sensor response of the coils is assumed. The maximum variation within the calibrated sensor response of the coils is 2.0%, whilst the standard variation is 0.9%. The precision of both the PCBs and magnetic coils is very high.

It can be that the sensors are precise and can measure a 50 Hz magnetic field accurately. Due to the constant underestimation with respect to the EHP-50, a calibration factor is added to the measured values of the sensor, which is valid for the entire measurement range. The underestimation could be due to cable loss of the coils and induced noise. The 0.4 µT range of the sensors are fully calibrated and can accurately measure the magnetic field.

The 100 µT range was calibrated with equal magnetic field strengths, as the current setup could not generate stronger fields. However, the higher range can still accurately measure lower field strengths, although the minimal step response is higher. The average variation of this range is in agreement with that of the 0.4 µT range. The higher magnetic field strengths are validated inside a power distribution sub-station (Section 3.3.3).

The sensor is calibrated within the following ranges:

- 0.4 µT range (0.08 µT to 58 µT), with a minimal single axis step response of 2 nT.
- 100 µT range (0.1 µT to 364 µT), with a minimal single step response 13 nT.

However, the used coil (MC858) has a reported measurement uncertainty of 5%. The EHP-50 also has a measurement uncertainty of 5% (95th confidence interval). Since these uncertainties are independent, our custom-developed sensor demonstrates a measurement uncertainty of 7% (95th confidence interval). This shows the accuracy of our sensor, given that the expensive baseline equipment also has a measurement uncertainty of 5%.

### 3.3. In-Field Validation

#### 3.3.1. Validation in Residential Area

A sensor was installed on the top floor of a house built under a 150 kV high-voltage line. Both sensors (EHP-50 and own sensor) were installed next to each other at a distance of 10 cm to limit interference. Ideally, the distance between the sensors is higher, but this was not practically possible. The distance to the source, high-voltage power line, was about 17 m. To ensure that all samples that were recorded, all measured data samples were locally stored on an internal SD card instead of being wirelessly transmitted to a backend. However, the PCB does not have a real-time clock, which is not needed because it can rely on the timestamps provided by the backend, there is no time information during these tests. Hence, the results are prone to drift with regard to the EHP-50. Furthermore, the code is built in such a way that during the writing of the values to the SD card, no measurements can be performed.

**Figure 5:**
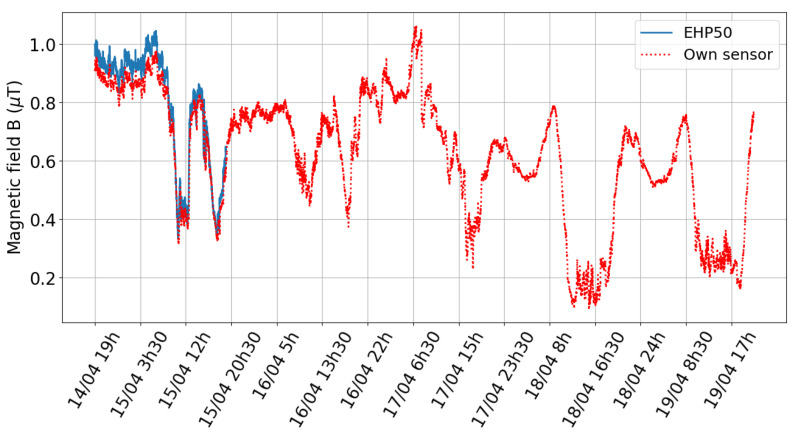
Measurement under high-voltage line from 14 April 2023 to 19 April 2023. The battery of the EHP-50 is limited to 24 h.

**Figure 6:**
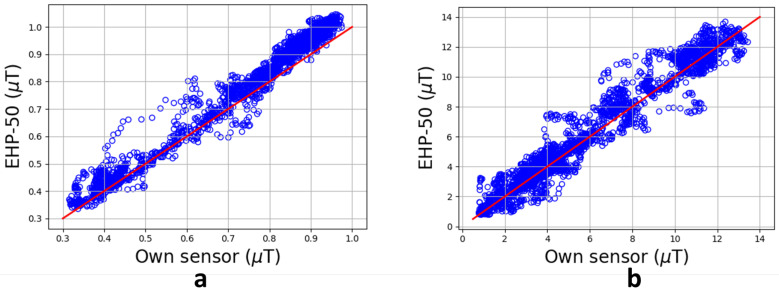
Relative deviation of the sensor with regard to the EHP-50. (**a**) Test in a residential area; (**b**) test in an industrial area. Blue circles: measured data, red line: expected line of regression.

Figure 5 shows the magnetic field under the high-voltage line between 14 April 2023 and 19 April 2023. The EHP-50 was able to measure for 24 h before the battery ran out. The magnetic field measured with the own developed sensor varies between 0.10 µT and 1.06 µT, measured within the 0.4 µT measurement range. Compared to the EHP-50, an average relative deviation of 6.2% is found, with a maximum of 31.9%. Figure 6a illustrates the deviation between both systems by means of a scatterplot of the recorded values. The red line indicates the expected result, and the blue dots represent the measurements. The deviation between both systems increased during the test as they had a separate internal clock due to the SD card which was desynchronized and thus influenced the result. Hence, due to the timing desynchronization, a high maximum deviation is obtained.

To verify that the timing desynchronization is responsible for the high maximum deviation, an additional test was performed on a different location in which the sensor sent the data to a backend. Hence, with the use of the timestamps, the data could now be synchronized. An average relative deviation of 5.2% is now obtained, with a maximum of 12.3%. This leads to the conclusion that the high deviation is caused by the desynchronization.

In general, an average difference of 5.8% is obtained, with a maximum of 7.9%. A Pearson correlation of 0.99 was found between both measurement systems.

The difference of 5.8% with respect to the benchmark could be originating from the calibration. In the calibration, the source is next to the sensor instead of above the sensor as the high-voltage power lines. Hence, it must be investigated if altering the source during the calibration could improve the results. Furthermore, an additional in-field calibration could be performed whilst installing the sensors, but this would be time-consuming.

#### 3.3.2. Validation in Industrial Area

An additional measurement has been performed under a 380 kV high-voltage line between 27 November 2023 and 28 November 2023 in an industrial area to compare the sensor to the EHP-50. This additional measurement was performed as higher magnetic field values were expected under this high-voltage line. The sensors were placed on the second story of the building to decrease the distance to the power line, which resulted in higher field values in comparison to the first floor.

The measured magnetic field varied between 0.77 µT and 13.43 µT. Compared to the EHP-50, an average relative deviation of 1.4% is obtained. Figure 6b shows the relative deviation of the sensor with regard to the EHP-50. The expected response is plotted in red, whilst the actual measurements are displayed in blue. An average underestimation of 3.3% is obtained. Both validations show a further underestimation compared to what was obtained during the in-lab calibration. A Pearson correlation of 0.97 was obtained between both measurement systems. Hence, it can be concluded that the developed sensor measured the magnetic field accurately.

Taking into account the EHP-50’s and our own sensors’ measurement uncertainty of at least 5%, it can be deduced that the new sensor is calibrated correctly and that it can measure 50 Hz magnetic fields originating from high-voltage lines accurately.

#### 3.3.3. Validation in Power Distribution Sub-Station

Measurements were performed inside a power distribution sub-station to verify the calibration and to ensure that this calibration is valid over the entire measurement range. However, due to the need for high fields, the sensors must be placed close (<30 cm) to the source. This makes a comparison between the EHP-50 and the own sensor more difficult. The sources were four (L1, L2, L3 and N) transport cables originating from a 12 kV transformer. The edge of our sensor was placed at a distance of 18 cm from cables L1 and L2, while the EHP-50 was placed at a distance of 15 cm from cables L2 and L3. However, the coils of the developed sensor are not positioned at the edge but are centered within the sensor, hence the distance towards the source increased even further.

Our own sensor measured a magnetic field between 29.2 µT and 113.3 µT. A comparison between the normalized values of EHP-50 and those of the developed sensor leads to an average relative deviation of 4.8%.

The designed sensor is now completely calibrated and verified. The accuracy of the sensor is within the measurement uncertainty of 7% with respect to the baseline, for both measurement ranges. The sensors are very precise, with only an average variation of 0.7% between the calibrated sensors. The sensitivity is high, and the field values of 0.08 µT to 364 µT can be measured, and combined with a high accuracy of 2 nT.

#### 3.3.4. Comparison of Measured Values to Literature

Tanaka et al. [25] reported a maximum field value of 2 µT for spot measurements performed with an EMDEX II under a 500 kV high-voltage power line. These measurements were performed in seven countries at 23 power facilities, from 1997 to 2001. Al-Bassam et al. [31] reported measurement performed in Kuwait in 2013 and 2014. A maximum field value of 14.79 µT was measured directly under a 300 kV line. Ozen [30] reported maximum field values of 3.3 and 4.3 µT for a 380 kV line and a 154 kV line in Antalya, Turkey. The measurements were performed in 2006. Nicolaou et al. [24] reported magnetic field values under 66 and 132 kV lines. For the 66 kV, a maximum field value of 1.985 µT was obtained. For the 132 kV line, a maximum field value of 2.108 µT was measured. All measurements were obtained in Cyprus. Merchant et al. [42] reports a survey of domestic power-frequency exposure for employees in the electricity supply industry in the UK between April 1989 and March 1992. These were given an exposimeter for a week. We selected the measurements for which the high-voltage lines within 100 m of these static monitors. A maximum field of 6.453 µT was obtained.

The field values obtained by the developed sensors were within 0.10 and 13.43 µT. These results are thus in agreement with the previous measured field values in the literature above. The maximum obtained field value of 13.43 µT is 6.7% of the current ICNIRP guideline and 13.4% of the Flanders and European Union guidelines.

## 4. Long-Term Monitoring

### 4.1. Sensor Tested during 118 Days

**Figure 7:**
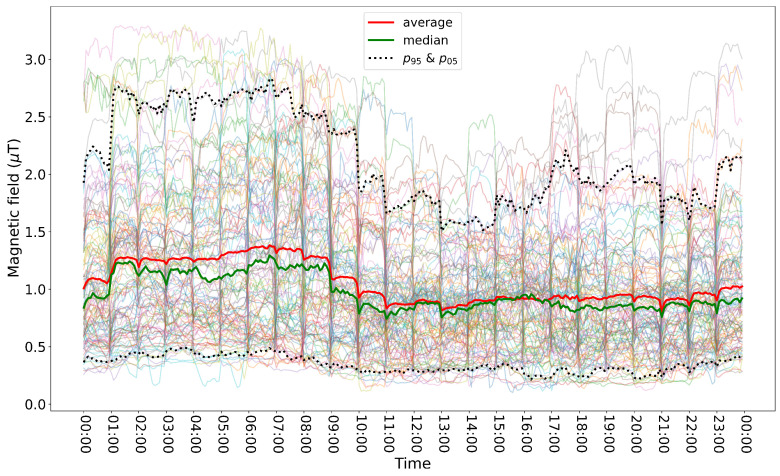
Overview of total measurement period from one sensor. Each line represents one day, 118 days are shown.

Four sensors have been deployed in the field in Flanders. First, the focus is on the earliest deployed sensor to determine any trends within the power. Later, in Section 4.2, the focus is on the capabilities of the sensors to form a monitoring network. The first sensor was installed directly under a 380 kV high-voltage line in an industrial area. A long-term test was performed, in which a total of 1.954.319 measurements were wirelessly collected through the sensor’s IoT communication (i.e., MQTT via 4G connection) from 12 September 2023 to 9 January 2024, which was 96% of all samples expected. Figure 7 shows these measurements in a timeframe of 24 h for each measurement day. A fixed temporal resolution of 5 min was used to construct this graph, i.e., each sample point is the average of 5 min of data. Each faded line corresponds to a measurement day (divided into 288 sample points). In total, 118 days are displayed. Figure 7 also shows the fifth and 95th percentile (p05 and p95, black dotted line), median (green line) and average (red line) of the measurement days. These are day-combined values of all 5-min bins.

The measured values varied between 0.13 µT and 3.83 µT. An average field value of 1.22 µT is obtained, whilst the median field value is 1.06 µT. All reported values satisfy the current ICNIRP guidelines (i.e., all field values are lower than 200 µT).

A high variability throughout the day can be observed in the daily data (i.e., faded lines). No clear daily pattern was distinguished. However, the average magnetic field is higher during the night (i.e., 1.49 µT between 1 h and 9 h) than it was during the day (i.e., 1.09 µT). This might be due to solar panels. This pattern is only valid for this specific high-voltage line. Based on 17 weeks of data, no pronounced difference was found in magnetic field strength during the weekdays vs the weekend.

**Figure 8:**
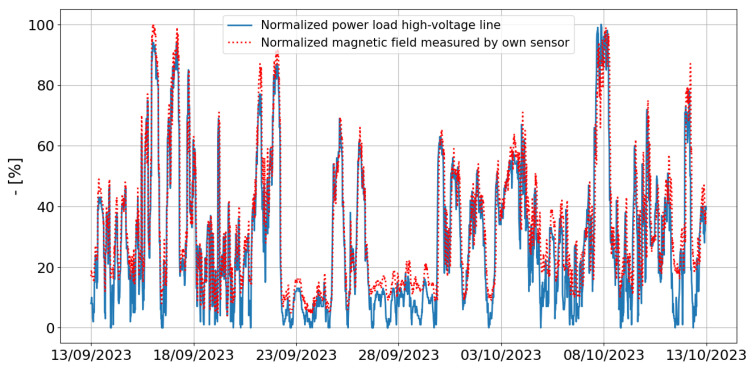
Comparison between normalized measured magnetic field from our developed sensor and power load data from the specific high-voltage power line.

Figure 8 compares the normalized magnetic field obtained by the new developed sensor to publicly available normalized load power data of the considered high-voltage line during 30 days from 13 September 2023 to 13 October 2023. The load power data are available in 15 min time intervals. Therefore, we rescaled our sensor data to a 15 min average magnetic field value, which is possible due to the backend, which allocates timestamps to the sent data. The load power varies between 0% and 41.1% of the maximum possible line loading. An excellent agreement and similar pattern is observed. The magnetic field values are related to the load power of the high-voltage line, especially for higher normalized power loads (>20%). For these field values, an average deviation between the measurement sensor and the available data is 5.5%. However, the load power is not the only predictor for the magnetic field, especially when the power was low (<20%, i.e., a load lower than 8.22% of the maximum possible line loading). Here, the average deviation increased. This could be due to another source close to the sensor. In this specific case, two high-voltage line are placed in parallel. Hence, if the load power is low in the high-voltage line under test and high in the parallel power line, the power line further could be the main source of exposure.

The load power of the high-voltage line can change rapidly. A maximum change of 47% was found for the observed time period. These sudden changes also occurred in the magnetic field, simultaneously with the load power. It can thus be concluded that our own developed sensor can measure 50 Hz magnetic fields originating from high-voltage power lines accurately.

### 4.2. Monitoring Network

**Figure 9:**
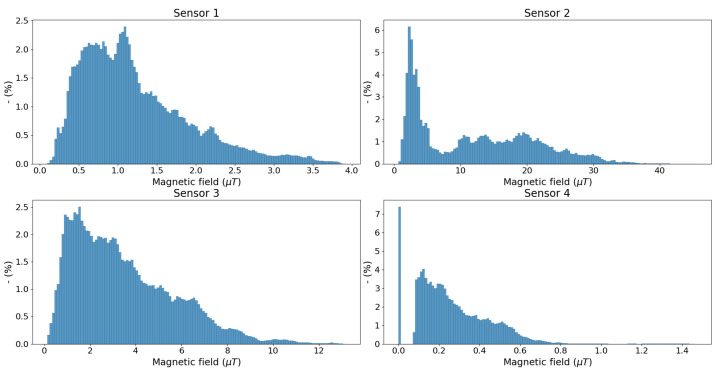
Distribution of the measured field values of the four considered sensors.

To verify the monitoring capabilities of the sensors, four sensors were placed in the field for at least three months. Sensors 1 and 3 are placed directly under a 380 kV high-voltage power line. Sensor 2 is placed above an underground 380 kV power line. Sensor 4 is placed at a distance of 50 m perpendicular to a 380 kV power line. In total, over 6 million samples were obtained by the four sensors in a period between 13 September 2023 and 15 February 2024. The fields ranged from 0.08 µT to 45.48 µT (Figure 9). Sensor 2 measured the highest fields (between 0.67 µT and 45.48 µT) as the distance between the source and the sensor is the smallest. Sensors 1 and 3 are placed directly under a different high-voltage power line and at different heights. Sensor 3 is placed on the second floor, while sensor 1 is placed on the ground floor. The magnetic field ranges between 0.1 µT and 13.1 µT. Sensor 4 measures the lowest magnetic fields (between 0.08 µT and 1.47 µT), which is expected as the sensor is placed at a distance of 50 m from the source. This case is particularly interesting because it can help governments impose safety distances when implementing precautionary measures for sensitive population groups (e.g., children), based on real, measured exposure. Sensor 4 also reported field values below the sensitivity of 0.08 µT, these values are set to zero. Up to 7.4% of the measured values were lower than the sensitivity.

For the four sensors, the uptime was 96%, 82%, 81% and, 95%. For the second sensor, an error was obtained within the processing unit during which the sensor was blocked. If the period of lock-up is omitted, an uptime of 95% is obtained. Similar results are obtained for the third sensor, where the lock-up also occurred. This lock-up most likely occurred due to a loss of connection. The software has been updated to prevent the lock-ups. Considering this is a prototype monitoring network, these results are really good. The result enables us to further roll-out of multiple sensors to test the monitoring network on a large scale with an increasing number of sensors.

## 5. Conclusions

A low-cost, tri-axial 50 Hz magnetic field monitoring sensor was designed, calibrated and verified. The sensor was designed using off-the-shelf components and commercially available coils. It can measure 50 Hz magnetic fields originating from high-voltage power lines from 0.08 µT to 364 µT, divided into two measurement ranges. A bandpass filter is constructed between 9.65 Hz and 318.31 Hz. In the software, an additional filter was added to obtain a small band sensor that focuses solely on 50 Hz. The sensor has an internal sampling rate of 2000 samples per second, whilst it wirelessly transmits to, and stores values in, an online database every 5 s.

The sensor was calibrated both on-board and in-lab. The on-board calibration takes the circuit attenuation, noise and parasitic components into account. In the in-lab calibration, the output of the developed sensor is compared to the benchmark, a narrowband EHP-50. The sensor was then verified in situ. The sensor was verified under both a residential and industrial high-voltage power line. It was compared to the EHP-50, for which average deviation of 6.2% and 1.4% were found, at two independent measurement locations. Fields up to 113.3 µT were measured in a power distribution sub-station to ensure that both measurement ranges were verified.

The measured field values were between 0.10 µT and 13.43 µT, which is in agreement with other reported measurement values under high-voltage power lines in the literature. Furthermore, all reported values satisfy the current ICNIRP guidelines (i.e., all field values are lower than 100 µT). Our network, four active sensors in the field, had high uptimes of 96%, 82%, 81% and, 95% during a minimum 3-month interval. In total, over 6 million samples were gathered. This suggests that the proposed solution can be used for this monitoring, although more extensive long-term testing with more sensors is required to confirm the uptime under multiple circumstances.

Future work will consist of the deployment of the sensors over a large area (e.g., Flanders) and under multiple high-voltage power lines. The analysis of the long-term monitoring of this deployment will be required. Furthermore, it will be investigated if the ELF sensor can be combined with another sensing technology (e.g., RF, noise).

## Figures and Tables

**Table 1:** Overview of commercially available ELF measurement systems.

Model	Frequency Range	Axis	Magnetic Field Measurement Range	Electric Field Measurement Range	Price	Additional Features
EHP-50	1 Hz–400 kHz	3	0.3 nT–10 mT	5 mV/m–100 kV/m	€€€€€	Datalogging up to 36 h
ELT-400	1 Hz–400 kHz	3	1 nT–320 µT OR 10 µT–80 mT	100 V/m OR 50 kV/m	€€€€	Comparison to standards on device
NFA30M	16 Hz–30 kHz	3	1 nT–20 µT	0.1–2000 V/m	€€	Datalogging up to 48 h, max hold
EMDEX II	40 Hz–800 Hz	3	0.01 µT–300 µT	/	€€€	Battery up to 7 days
Extech 480823	30 Hz–300 Hz	1	0.01 tot 20 µT	/	€	Hand-held device
Tenmars TM-190	50/60 Hz	3	0.01 µT–20 µT OR 0.1 µT–200 µT	1 V/m–2000 V/m	€	Can measure ELF and RF
Tenmars TM-191	30 Hz–300 Hz	1	0.01 µT–20 µT OR 0.1 µT–200 µT	/	€	Hand-held device, max hold
Tenmars TM-192D	30 Hz–2kHz	3	0.001 µT–2 µT OR 0.01 µT–20 µT OR 0.1 µT–200 µT	/	€	Hand-held device, max hold 9999 data logs
***Proposed sensor, minimal requirements***	***10 Hz–300 Hz***	***3***	***0.1 µT to 200 µT***	***/***	***€/€€***	***IoT connectivity long-term datalogging***

**Table 2:** In-lab calibration. Twelve sensors were calibrated at distance (Test 1 d = 0.5 m; test 2 d = 1.75 m) from the source.

	Test 1 (µT)	Deviation w.r.t. EHP-50 Test 1 (%)	Test 2 (µT)	Deviation w.r.t. EHP-50 Test 2 (%)
EHP-50	30.94		1.66	
SENSOR01	19.47	62.9	1.06	63.4
SENSOR 02	19.58	63.3	1.06	63.6
SENSOR 03	19.61	63.4	1.06	63.4
SENSOR 04	19.75	63.8	1.05	63.4
SENSOR 05	19.65	63.5	1.06	63.6
SENSOR 06	19.69	63.6	1.05	63.1
SENSOR 07	19.45	62.9	1.05	63.0
SENSOR 08	19.61	63.4	1.06	63.4
SENSOR 09	19.53	63.1	1.05	63.1
SENSOR 10	19.57	63.2	1.05	63.1
SENSOR 11	19.67	63.6	1.06	63.7
SENSOR 12	19.51	63.0	1.06	63.7

## Data Availability

The raw data supporting the conclusions of this article will be made available by the authors on request.
